# Electrocardiologic and Echocardiographic Findings in Patients With Scorpion Sting

**DOI:** 10.5812/ircmj.2853

**Published:** 2013-05-05

**Authors:** Ahmadnoor Abdi, Hossein Farshidi, Shafei Rahimi, Abdulrahim Amini, Sayedeh Fatemeh Tasnim Eftekhari

**Affiliations:** 1 Cardiovascular Research Center, Hormozgan University of Medical Sciences, IR Iran; 2 Infectious Diseases Research Center, Hormozgan University of Medical Sciences, IR Iran

**Keywords:** Scorpions, Electrocardiography, Echocardiography

## Dear Editor,

Scorpion sting is a major public health problem in many parts of the world. High prevalence, severity of symptoms and difficulty of treatment are problems of scorpion sting (1). This problem is more prevalent in tropical and subtropical areas. These areas include the North-Saharan African, Sahelian Africa, South Africa, the Middle East, southern India, Mexico, Latin America and the Andean region (2). The most acceptable hypotheses for pathogenesis of cardiac damage secondary to scorpion bites are increase catecholamine due to the direct stimulatory effect of scorpion venom on the adrenal glands or a direct sympathomimetic cardiac effect of the venom (3). ECG changes resulting from scorpion stings maybe recorded on admission or several hours later. These changes could have a very wide range. Earliest recorded findings are sinus tachycardia. However, the percentage of patients with bradycardia may also be present (4). Changes in echocardiography could include reduced left ventricular function, enlarged left ventricle and reduced end diastolic volume (EDV), reduced end systolic volume (ESV) and reduced ejection fraction (EF) (5). The aim of the present study is examine the electrocardiography and echocardiography changes in patients presenting with envenoming following a scorpion sting. Forty-three patients, who had admitted to Shahid Mohammadi Hospital (General teaching hospital in Bandar Abass, Iran) due to scorpion sting between March and September 2008, were included to this cross sectional study. Patients, who had history of heart diseases, hypertension, diabetes mellitus, over 60 years old and unknown bite were excluded. Patients were assigned to one of three clinical states, mild, moderate and severe (6). Standard 12 lead electrocardiography and trans-thoracic echocardiography (M-Mode and B-Mode) were performed for patients. Electrocardiography was performed at admission time and every 6-hour. Echocardiography within 24 hours after admission were performed. Echocardiographic parameters that were evaluated that were included End Systolic Diameter (ESD), End Diastolic Diameter (EDD), EF, hypertrophy, regional wall motion abnormalities (RWMA) and the pericardium. Epi Info software (V.3.5.1 for widows) is used for statistical analysis. The data were analyzed using student’s t-test and chi-square test. P values of < 0.05 were considered statistically significant. Forty-three patients were included this analysis. The average age of the patients was 27.11 ± 10.10 years and 90% of patients were under 45 years (Table 1). Odontobuthus doriae (Buthidae) is dominant species in hormozgan provices (location of study) but we had not available to scorpion with patient for diagnosis species of scorpion. Twenty-seven patients (62.8%) were classified as mild clinical state, 14(32.5%) as moderate and two (4.7%) as severe. Thirteen patients (30.2%) had abnormal ECGs. We did not fine a significant association between frequency of abnormal ECGs and the severity of clinical findings (P = 0.0587).

**Table 1.** Distribution of PatientsWith Normal and Abnormal ECGs

	Total	Abnormal ECG	Normal ECG	P value ^a^
**Number, No.** **(%)**	43(100)	13(30.2)	30(69.8)	
**Gender** **, No. (%)**				0.4597
Male	21(48.8)	7(53.8)	14(46.7)	
Female	22(51.2)	6(46.2)	16(53.3)	
**Age(y), Mean ± SD**	27.11±10.10	23.92±6.11	28.50±11.22	0.1756
**Site of bite, No. (%)**				0.3442
Upper limbs	18(52.9)	6(54.5)	12(52.2)	
Lower limbs	13(38.2)	3(27.3)	10(43.5)	
Body Head and Neck	3(8.9)	2(18.2)	1(4.3)	
**Clinical findings state, No. (%)**				0.0597
Mild	27(62.8)	6(46.1)	21(70)	
Moderate	14(32.5)	5(38.5)	9(30)	
Severe	2(4.7)	2(15.4)	0(0)	

^a^P values of < 0.05 were considered statistically significant

The most common abnormality in ECG was PVC (13.9%) and others were including ST depression (9.3%), T-inversion (4.6%), AF (4.6%), U-wave (2.3%) and sinus arrhythmia (2.3%). There is no case of atrial-ventricular blocks. There are 5 cases (11.6%) of sinus tachycardia and one case of sinus bradycardia. The patients were evaluated with echocardiography in the first 24 hours of admission. ESD, EDD, and EF were in normal range in all patients. None of the patients had evidence of hypertrophy, RWMA and pericardial disorders. Although, we did not observe statistically significant relationship between severity of clinical symptoms and electrocardiographic changes but 100% of patients with severe symptoms, 35.7% of patients with moderate symptoms and 22.2% patients with mild symptoms had been admitted with electrocardiographic change. In this study, no death was observed and all patients were discharge with good outcome. Physicians who working in endemic areas should be informed of scorpion bites and potentially dangerous side effects caused by common species. It is impotence to selecting the appropriate protocol to treat victims. The findings in recent study show that 30.2% of patients with scorpion stings have been at least an abnormal ECG changes, such as PVC, PAC, AF, T wave and ST segment changes. These heart complications can be cause death. In our study, the most common ECG finding was PVC (13.9% of all patients). In addition, we fine PAC in 11.6% of patients. Previous studies show that PVC, LBBB, atrial tachycardia, incomplete blockage and PAC are commonly findings in patients with scorpion bite (4, 6). The recent study, only five patients (11.6%) had sinus tachycardia and one case (2.3%) had sinus bradycardia. Sinus tachycardia is early findings therefore delay in referring patients to hospital maybe the cause of lower incidence of sinus tachycardia in this study. In the recent study, all patients had normal echocardiography findings. Although, there was no significant correlation between the prevalence of electrocardiographic findings and clinical symptoms, but the prevalence of electrocardiography findings in patients with mild, moderate and severe clinical symptoms were 22.2%, 35.7% and 100%, respectively. According to findings of our study, cardiac disorders following scorpion stings have high prevalence. Therefore, it is important that patients be presented with scorpion sting are evaluated with electrocardiography and heart monitoring, particularly in patients with severe symptoms should be used. Echocardiography for all patients is not economically but electrocardiography is cost-effective and justified.

## References

[1] Chippaux JP, Goyffon M (2008). Epidemiology of scorpionism: a global appraisal.. Acta Trop..

[2] Otero R, Navio E, Cespedes FA, Nunez MJ, Lozano L, Moscoso ER (2004). Scorpion envenoming in two regions of Colombia: clinical, epidemiological and therapeutic aspects.. Trans R Soc Trop Med Hyg..

[3] Gueron M, Yaron R (1970). Cardiovascular manifestations of severe scorpion sting. Clinicopathologic correlations.. Chest..

[4] Alan S, Ulgen MS, Soker M, Geyik F, Karabulut A, Toprak N (2004). Electrocardiologic and echocardiographic features of patients exposed to scorpion bite.. Angiology..

[5] Kumar EB, Soomro RS, al Hamdani A, el Shimy N (1992). Scorpion venom cardiomyopathy.. Am Heart J..

[6] Diaz P, Chowell G, Ceja G, D'Auria TC, Lloyd RC, Castillo-Chavez C (2005). Pediatric electrocardiograph abnormalities following Centruroides limpidus tecomanus scorpion envenomation.. Toxicon..

